# Youth’s climate consciousness: unraveling the Dengue-climate connection in Bangladesh

**DOI:** 10.3389/fpubh.2024.1346692

**Published:** 2024-06-12

**Authors:** Abu Bakkar Siddique, Maruf Hasan, Ayesha Ahmed, Md Hafizur Rahman, Md Tajuddin Sikder

**Affiliations:** ^1^Department of Public Health and Informatics, Jahangirnagar University, Savar, Dhaka, Bangladesh; ^2^Centre for Advanced Research Excellence in Public Health, Savar, Dhaka, Bangladesh; ^3^International Centre for Research, Innovation, Training and Development (ICRITD), Dhaka, Bangladesh; ^4^AMR Reference Laboratory (Research), Bangladesh Livestock Research Institute, Savar, Dhaka, Bangladesh; ^5^Health and Environmental Epidemiology Laboratory (HEEL), Jahangirnagar University, Savar, Dhaka, Bangladesh

**Keywords:** dengue, knowledge, perception, climate change, youth, Bangladesh

## Abstract

**Background:**

Climate change affects the transmission of vector-borne diseases like dengue, posing a substantial public health threat. Bangladesh, with its favorable conditions for Dengue transmission, has experienced periodic outbreaks. This study explores the relationship between climate change knowledge, perceptions of the Dengue-climate link, and the associated factors among Bangladeshi youth.

**Methods:**

In the Dhaka district of Bangladesh, a cross-sectional study was conducted between September and October 2023, involving face-to-face interviews with 1,358 participants. Convenient (non-probability) sampling was utilized for participant selection. Data collection involved the administration of a semi-structured questionnaire encompassing informed consent, socio-demographic information, and inquiries pertaining to climate change-related knowledge (13 items) and perception (11 items). Data analysis utilized STATA (Version 15.0) and SPSS (Version 26.0).

**Results:**

The mean scores for knowledge and perceptions were determined to be 7.10 ± 3.20 (out of 13) and 26.60 ± 4.12 (out of 33) respectively. Participants had a mean age of 22.02 ± 1.58 years. The study revealed that unmarried status, living in a nuclear family, being a non-smoker, good self-perception of physical health, regular sleep patterns, moderate social media usage, older age, unemployment, and daily media consumption are factors associated with higher knowledge and perception regarding the Dengue-climate change link. Moreover, a positive association was observed between knowledge of climate change and favorable attitudes toward the Dengue-climate connection.

**Conclusion:**

This study underscores the importance of tailored climate change education for youth in Bangladesh and highlights key variables influencing their knowledge and perceptions. Notably, there exists a positive association between climate change knowledge and favorable attitudes toward the Dengue-climate connection. These insights underscore the importance of targeted educational campaigns and policy interventions aimed at enhancing climate consciousness among the youth population, thereby fostering proactive measures to mitigate the impact of Dengue fever in the context of climate change.

## 1 Introduction

Climate change refers to long-term shifts in temperatures and weather patterns (1). In recent decades, there has been a discernible pattern of temperature change. Overall, temperatures have exhibited a gradual upward trend, indicating a long-term warming of the climate (2). This trend has been observed across many parts of the world, with variations in the rate and intensity of warming in different regions (3). Moreover, climate change affects most of the organism’s life including their habitual status and life cycle (4).

Climate influences the transmission patterns, geographic distribution, and resurgence of vector-borne illnesses via various mechanisms, impacting pathogen, vector, non-human host, and human populations directly (5). Furthermore, climate change can modify entire ecosystem environments, encompassing urban habitats, potentially affecting the suitability of habitats for vectors and non-human hosts (6, 7). In the case of Dengue, an increase in temperature can significantly elevate the transmission of Dengue by directly affecting both the *Aedes* mosquito vector and the Dengue virus. Elevated temperatures expedite the life cycle of mosquitoes, resulting in heightened breeding rates, shorter virus incubation periods within mosquitoes, and an increased probability of successful viral transmission to humans (8). Moreover, rising temperatures enhance the mosquito’s activity and feeding patterns, resulting in more frequent and efficient biting encounters with humans (9). This combination of factors, driven by warmer conditions, creates a conducive environment for the Dengue virus to thrive and spread, amplifying the risk of Dengue transmission in regions experiencing temperature increases due to climate change (10).

**Figure 1:**
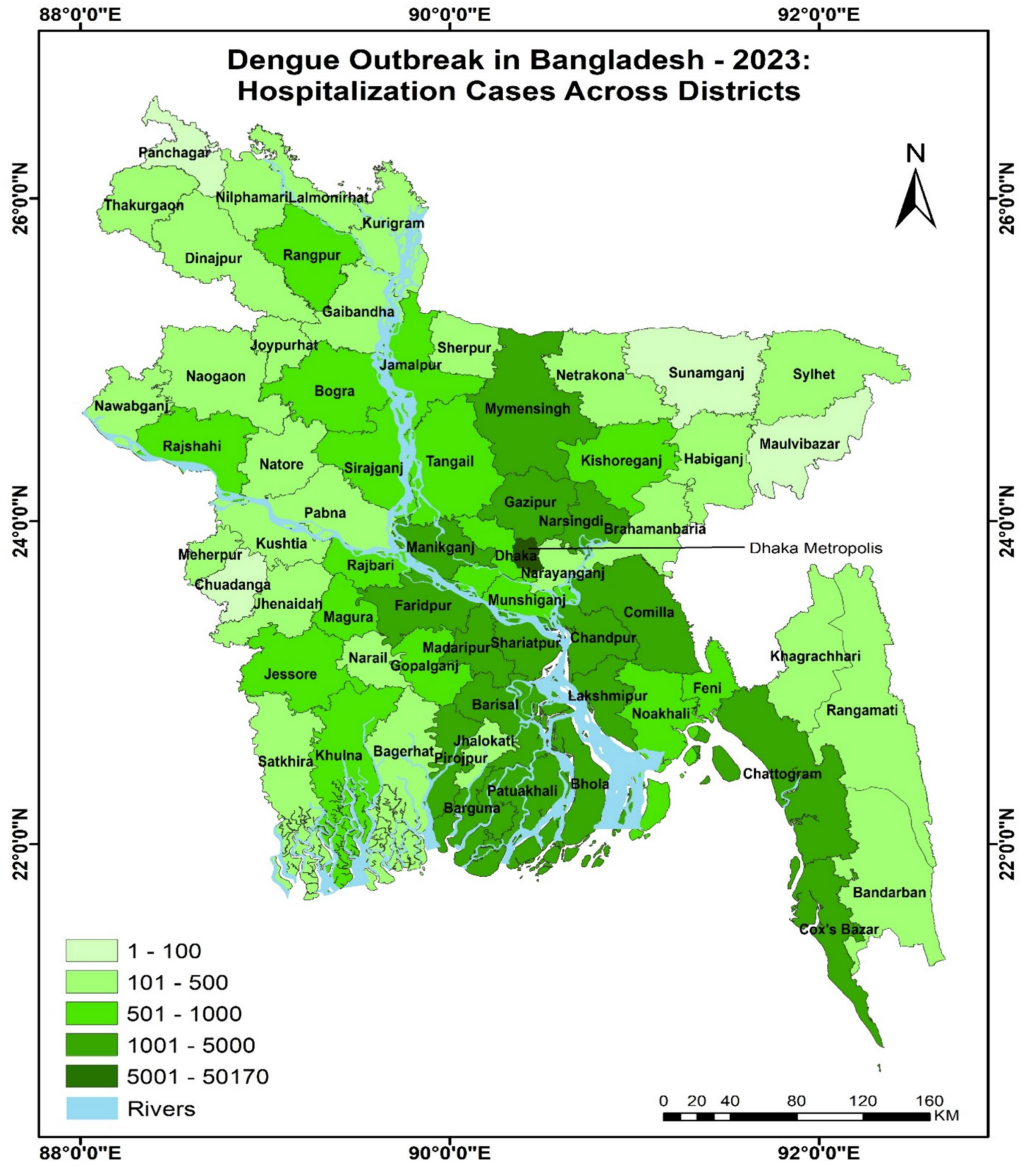
Dengue outbreak in Bangladesh–2023.

Dengue stands as a significant threat among vector-borne diseases, claiming approximately 40,500 lives globally, with a majority of fatalities occurring in Asian countries (11). With 3.5 billion individuals residing in Dengue-endemic regions worldwide and facing the risk of infection, 1.3 billion people inhabit Dengue-endemic areas across ten countries in the South East Asia (SEA) Region. Alarmingly, dengue cases in the SEA region surged by 46% from 2015 to 2019, escalating from 451,442 to 658,301 cases during this period (12, 13). Additionally, the prevalence of Dengue in sub-tropical regions, including Bangladesh, has been a growing concern due to the conducive climate conditions that favor the *Aedes* mosquito vector and the Dengue virus. In recent years, Bangladesh has experienced periodic Dengue outbreaks underscore the critical need to address the disease in this sub-tropical region. According to data up to 27 August 2023, a significant number of cases—119,133 in total—and 569 deaths have been recorded across 64 districts in Bangladesh this year (14). Among the major cities in Bangladesh, Dhaka is the riskiest area of dengue disease present in Bangladesh according to the director general of health services in Bangladesh (15). The distribution of dengue cases among different districts is shown in Figure 1.

From the above discussion, we can say that there is a strong relationship between climate change and Dengue prevalence (16). Moreover, the strong relationship between climate change and Dengue prevalence underscores the critical need for proactive measures to mitigate the impacts of a changing climate on public health and reduce Dengue prevalence (9). So, study about the relationship between climate change and dengue occurrence is very important nowadays.

Additionally, Dengue affects almost people of all ages, including substantial amount of death among youth in recent past years (17). Furthermore, Bangladesh’s youth constitutes a pivotal demographic cohort (18). Given that a significant proportion of the country’s populace belongs to this group, the future trajectory of the nation hinges significantly upon their actions. These young individuals will emerge as the architects of tomorrow, influencing policies, science, and advocacy efforts, thereby molding Bangladesh’s approach toward addressing climate change (19). So, it’s very important to know their real scenario currently.

This study aims to assess the level of knowledge concerning climate change and perceptions of the relationship between dengue and climate change among the youth in Bangladesh, along with the associated factors contributing to higher scores. To date, no study has concurrently examined knowledge related to climate change and the perception of the connection between dengue and climate change simultaneously in Bangladesh. Its importance lies in bridging knowledge gaps concerning “climate change” and “the correlation between climate change and dengue prevalence” among youth in Bangladesh. Through assessing the understanding of “climate change” and perceptions regarding “the link between dengue and climate change” among youth, this research aims to inform policy-making processes and shape the creation of tailored interventions to bolster climate awareness and dengue prevention strategies among youth populations. Ultimately, this research contributes to building a more informed and proactive approach toward addressing the complex challenges posed by climate change and its impact on public health, particularly regarding vector-borne diseases like dengue, in the context of Bangladesh and similar regions globally.

## 2 Materials and methods

### 2.1 Study area

The study was carried out at Dhaka district in Bangladesh by face-to-face interview based cross-sectional survey. Data was collected between September and October 2023.

### 2.2 Sample size

The sample size was calculated using the following equation:


$$n=\frac{z^{2}pq}{d^{2}};\;n=\frac{1.96^{2}\times 0.5\times \left(1-0.5\right)}{0.05^{2}}=384.16\approx 384$$

Here,

*n* = number of samples.

*z* = 1.96 (95% confidence level).

*p* = prevalence estimate (50% or 0.5), as no previous study found in Bangladesh.

*q* = (1-*p*).

*d* = Precession of the prevalence estimate (10% of 0.05).

In anticipation of the study, we hypothesized a prevalence estimate (*p*) of 50%. Accounting for a 10% non-response rate, we estimated a sample size of approximately 424 individuals, which was surpassed by the actual recruitment of 1,358 participants to bolster the robustness of our study.

### 2.3 Study design, participants, and procedure

The present study employed a cross-sectional survey design, utilizing face-to-face interviews with questionnaires, conducted over the span of September to October 2023. Participants were recruited through non-probability sampling (specifically convenient sampling) (20). Each interview session lasted approximately 10–15 min. Initially, 1,410 individuals participated in the surveys, but after filtering incomplete responses, the final analysis included 1,358 surveys. Data collection utilized a paper-based semi-structured questionnaire administered in Bangla, the participants’ native language, at their current residences. To ensure sensitivity and confidentiality, trained research assistants conducted data collection, maintaining strict confidentiality protocols. Our supervisor, an expert authority in this field, ensured the quality and rigor of this research at every stage.

A preliminary trial involving 30 participants from the target population was conducted to assess the questionnaire’s acceptability and clarity. Minor modifications were made to the questionnaire based on the feedback received during this pilot test. However, data from this preliminary trial were excluded from the final analysis. Additionally, the questionnaire commenced with an informed consent statement on its first page, outlining the study’s objectives, procedures, and participants’ right to refuse participation. Prior to commencing the survey, participants were required to give informed consent (i.e., *“Would you like to engage in this study of your own accord and willingly?”*). Participants meeting the following criteria were included in the study: (i) age between 15 to 24 years (youth) (21), (ii) proficiency in spoken and written Bengali, (iii) voluntary participation, and (iv) residency in Bangladesh. Individuals under 18 years old or over 24 years old were excluded from the interview process.

### 2.4 Measures

#### 2.4.1 Socio-demographic measures

Socio-demographic data collection included inquiries regarding age, marital status (married or unmarried), and educational attainment (below university level or at university level) (20), employment status (employed [any type of employment like job, business, freelancing, etc.]/unemployed) (22), place of residence (rural/urban/ semi-urban), monthly family income (less than 20,000 Bangladeshi Taka [BDT]/2000BDT to 30,000 BDT/more than 30,000 BDT) (23), gender (male/female), family type(nuclear/large), previous history of Dengue (yes/no), family history of Dengue (yes/no), previous history of vector borne disease (yes/no), daily average sleeping time (less than 7 h/7 to 9 h/more than 9 h), smoking status (yes/no), Daily newspaper reading/ bulletin watching (yes/ no), father’s occupation (job holder/businessman/others/unemployed), self-perception about own physical health (good/bad) (23).

#### 2.4.2 Knowledge regarding climate change and perception toward linkage between dengue and climate change

In this study, we utilized a questionnaire comprising 13 items assessing climate change-related knowledge and 11 items gaging perceptions of Dengue and climate change. These items were selected based on an extensive review of existing literature in the field (2–5, 24, 25). The skewness and kurtosis of the total scores fell within the range of ±2.

Participants were presented with a set of 13 questions designed to assess their knowledge of climate change, each offering three response options, such as ‘yes,’ ‘no,’ or ‘do not know.’ [e.g., “*Is there too much greenhouse gas (GHG) in the atmosphere? Does too much infrared (IR) radiation stay on Earth*?”] (see details in Table 1). In the analysis, affirmative (yes) responses were assigned a code of “1,” while negative responses (no) and responses indicating uncertainty (do not know) were assigned a code of “0 (26). The total score was calculated by adding up the scores of all items, resulting in a range from 0 to 13. A higher total score reflects a greater level of knowledge on the subject. The Cronbach Alpha of knowledge items were 0.86 indicating that there is a high internal consistency (27).

**Table 1:** Items of knowledge and perception.

	Items: knowledge toward climate change	Mean ± SD
1.	Is there too much greenhouse gas (GHG) in the atmosphere compared to normal range?	0.74 ± 0.43
2.	Does too much infrared (IR) radiation stay on Earth compared to normal range?	0.38 ± 0.48
3.	Can fossil fuels such as oil and coal usage increase greenhouse gas?	0.69 ± 0.47
4.	Do purchased products generate greenhouse gasses (GHGs)?	0.51 ± 0.50
5.	Are nitrogen oxides released from fertilizers?	0.51 ± 0.50
6.	Do landfills produce methane?	0.49 ± 0.50
7.	Is there too much consumption of milk and dairy products?	0.56 ± 0.49
8.	Does the Earth have an ozone hole?	0.57 ± 0.49
9.	Is emitted IR radiation absorbed by GHGs?	0.43 ± 0.59
10.	Are Carbon dioxide (CO_2_), Methane (CH_4_) and nitrous oxide (N_2_0) are all greenhouse gasses?	0.68 ± 0.46
11.	Climate change can be identified by changes in the mean or variability of its properties persisting for an extended period, typically decades or longer.	0.57 ± 0.49
12.	Do changes in temperature and precipitation patterns influence the spread of vector-borne diseases like dengue?	0.58 ± 0.49
13.	Is water vapor a greenhouse gas?	0.46 ± 0.49
	Items: perception toward dengue and climate change-related question	Mean ± SD
1.	Climatic elements, such as temperature and rainfall, affect the environments and life cycles of Dengue vectors.	2.56 ± 0.70
2.	There is a strong connection between climate changes and the risk of Dengue outbreaks.	2.46 ± 0.60
3.	There are patterns between changes in temperature and the occurrence of Dengue cases in various regions.	2.20 ± 0.74
4.	Changes in rainfall patterns affect the prevalence of Dengue outbreaks.	2.58 ± 0.65
5.	There are changes in Dengue outbreaks during rainy seasons nowadays.	2.50 ± 0.67
6.	Changes in climate conditions impact the transmission of Dengue.	2.47 ± 0.66
7.	Understanding climate’s influence on Dengue can help in developing effective prevention strategies.	2.46 ± 0.68
8.	There is sufficient importance of public awareness regarding the link between climate change and Dengue outbreaks.	2.53 ± 0.65
9.	Changes in humidity levels contribute to the spread of Dengue outbreaks.	2.41 ± 0.69
10.	The general public bears the primary brunt of the alterations in Dengue outbreaks resulting from climate change.	2.20 ± 0.74
11.	Governments should implement policies and strategies to address the health impacts of climate change, including Dengue.	2.24 ± 0.75

To evaluate perceptions regarding the connection between Dengue and climate change, a questionnaire comprising 11 questions was employed, utilizing a three-point Likert scale (e.g., 1 = disagree, 2 = neutral, 3 = agree) (26). Examples of such questions include: “*Climatic elements, such as temperature and rainfall, affect the environments and life cycles of Dengue vectors, There is a strong connection between climate changes and the risk of Dengue outbreaks*,” (see details in Table 1). The total score ranged from 11 to 33, calculated by adding up the scores of all items. A higher score reflected a more pronounced presence of positive attitudes. The Cronbach Alpha of attitudes items were 0.82 were 0.86 indicating that there is a high internal consistency (27).

### 2.5 Statistical analysis

The data underwent analysis utilizing Statistical Package for Microsoft Excel (version 2021), SPSS version 26.0 (Chicago, IL, USA), and STATA (version 15.0). Microsoft Excel facilitated cleaning, coding, and sorting processes. Subsequently, the Excel file was imported into SPSS for computation of descriptive statistics, encompassing frequencies, percentages, means, and standard deviations. Finally, bivariate and multivariable linear regression analyses, incorporating total scores of knowledge and perception measures, were conducted using STATA. A significance level of *p* < 0.05 was applied across all analyses.

### 2.6 Ethics statement

The research protocol underwent review and approval by the Biosafety, Biosecurity, and Ethical Clearance Committee at Jahangirnagar University, Savar., Dhaka-1342, Bangladesh [Ref. No: BBEC, JU/M2023/08(59)]. All procedures adhered to human research guidelines, including those outlined in the Helsinki Declaration. Prior to participation, informed written consent was obtained from each participant, detailing the study’s procedures, objectives, and assurance of confidentiality regarding their information. Data collection was conducted anonymously and analyzed using numerical codes.

## 3 Result

### 3.1 General characteristics of the participants

Table 2 provides an overview of the general characteristics of the population (*N* = 1,358) under study. The maximum percentage for each variable is as follows: for educational qualification, the majority have below university qualifications (50.2%), while almost an equal percentage have a university-level education (49.8%). In terms of permanent residence, the majority reside in urban areas (47.2%), followed by rural (34.4%) and semi-urban (18.4%). Concerning monthly family income, the highest percentage falls within the 20,000 to 50,000 BDT range (51.5%). Gender distribution is nearly equal, with 50.2% being male and 49.8% female. The majority are unmarried (92.6%) and live in nuclear families (50.5%). Employment status shows that most are unemployed (82.7%). About a quarter of the population has a previous history of Dengue (24.2%), while slightly more have a family history of dengue (26.1%). A similar percentage has a previous history of vector-borne diseases (26.1%). The largest group in terms of average sleeping time falls within the 7–9 h range (49.3%), and the majority do not smoke (79.7%). Around 42.6% engage in daily newspaper reading or bulletin watching. Regarding fathers’ occupation, job holders are the largest group (46.5%). Lastly, a significant proportion perceives their own physical health as good (86.8%). The mean age of the participants was (22.02 ± 1.58).

**Table 2:** General characteristics of the population (*N* = 1,358).

Variables	*n* (%)
Age (Mean ± SD)	22.11 ± 1.72
Educational qualification	
Below university	682 (50.2)
University level	676 (49.8)
Permanent residence	
Rural	467 (34.4)
Urban	641 (47.2)
Semi-urban	250 (18.4)
Monthly family income	
Less than 20,000 BDT	404 (29.8)
20,000 to 50,000 BDT	700 (51.5)
More than 50,000 BDT	254 (18.7)
Gender	
Male	682 (50.2)
Female	676 (49.8)
Marital status	
Unmarried	1,257 (92.6)
Married	101 (7.4)
Family type	
Nuclear family	686 (50.5)
Large family	672 (49.5)
Employment status	
Employed	235 (17.3)
Unemployed	1,123 (82.7)
Previous history of dengue	
Yes	328 (24.2)
No	1,030 (75.8)
Family history of dengue	
Yes	354 (26.1)
No	1,004 (73.9)
Previous history of vector-borne disease	
Yes	320 (26.1)
No	1,038 (76.4)
Average sleeping time	
Less than 7 h	595 (43.8)
7–9 h (normal)	669 (49.3)
More than 9 h	94 (6.9)
Smoking status	
Yes	275 (20.3)
No	1,083 (79.7)
Daily newspaper reading/ bulletin watching	
Yes	578 (42.6)
No	780 (57.4)
Father’s occupation	
Job holder	632 (46.5)
Business	452 (33.3)
Others	225 (16.6)
Unemployed	49 (3.6)
Self-perception about own physical health	
Good	1,179 (86.8)
Bad	179 (13.2)

BDT Bangladeshi Taka, 1 BDT 0.0091 U$$ in 20 November, 2023.

### 3.2 Knowledge regarding climate change

The average score for knowledge items was 7.10 (SD = 3.20) out of 13, reflecting an overall correct percentage of just 53.84%. According to multiple linear regression analysis, factors positively associated with the knowledge score comprised: (i) being unmarried (*ꞵ* = 0.07, *p* < 0.004) in reference to ‘married’, (ii) resided in a nuclear family (*ꞵ* = 0.06, *p* = 0.004) in reference to ‘large family’, (iii) non-smoker (*ꞵ* = 0.07, *p* = 0.008), (iv) having good self-perception about own physical health (*ꞵ* = 0.30, *p* = <0.001), (v) having bad self-perception about own physical health (*ꞵ* = 0.11, *p* = 0.008), (vi) having normal sleeping time (*ꞵ* = 0.27, *p* = <0.001), and (vii) 2 to 4 h of social media usage time (*ꞵ* = 0.06, *p* = 0.019)(Table 3).

**Table 3:** Regression analysis predicting knowledge regarding climate change.

Variables	Overall	Bivariable regression analysis					Multivariable regression analysis				
	Mean (SD)	*B*	SE	*t*	*β*	*p*-value	*B*	*SE*	*t*	*β*	*p*-value
Age		−0.03	0.07	−0.42	−0.01	0.672	0.03	0.08	0.42	0.01	0.674
Education level												
Below university	7.71 (3.35)	Ref.									
University	7.05 (3.06)	0.02	0.25	0.10	<0.01	0.922	−0.05	0.26	−0.23	<0.01	0.820
Permanent residence											
Rural	6.96 (3.35)	Ref.					Ref.				
Urban	7.26 (3.15)	0.31	0.28	1.10	0.03	0.270	0.27	0.30	0.91	0.02	0.361
Semi-urban	7.00 (3.07)	0.20	0.36	0.56	0.01	0.573	0.16	0.36	0.45	0.01	0.653
Monthly family income											
> 20,000 BDT	7.23 (3.25)	0.12	0.37	0.35	0.05	0.730	0.49	0.40	1.22	0.04	0.221
20,000–30,000 BDT	7.00 (3.26)	−0.21	0.33	−0.63	0.03	0.531	−0.13	0.34	−0.38	0.03	0.704
> 30,000 BDT	7.23 (2.99)	Ref.									
Gender											
Male	7.04 (3.37)	Ref.					Ref.				
Female	7.18 (3.03)	0.36	0.25	1.44	0.03	0.151	0.24	0.27	0.91	0.02	0.362
Marital status											
Unmarried	7.16 (3.16)	1.36	0.47	2.85	0.07	**0.004**	1.13	0.49	2.29	0.06	**0.022**
Married	6.46 (3.74)	Ref.					Ref.				
Family type											
Nuclear	7.38 (3.19)	0.57	0.25	2.27	0.06	**0.023**	0.45	0.20	2.10	0.04	**0.045**
Large	6.83 (3.21)	Ref.					Ref.				
Employment status											
Employed	7.14 (3.23)	Ref.					Ref.				
Unemployed	7.10 (3.20)	0.49	0.23	1.50	0.04	0.135	0.22	0.34	0.64	0.01	0.524
Previous history of dengue											
Yes	7.08 (3.14)	Ref.					Ref.				
No	7.12 (3.23)	0.35	0.29	1.19	0.03	0.233	0.21	0.37	0.57	0.10	0.568
Family history of dengue											
Yes	7.10 (3.12)	0.39	0.28	1.38	0.03	0.168	−0.28	0.31	−0.91	−0.02	0.365
No	7.11 (3.24)	Ref.					Ref.				
Previous history of vector borne disease											
Yes	7.21 (3.04)	0.00	0.29	0.02	0.11	0.983	0.38	0.36	1.08	0.03	0.282
No	7.08 (3.26)	Ref.					Ref.				
Average sleeping time											
Less than 7 h	7.03 (2.98)	0.14	0.51	0.28	0.01	0.780	−0.07	0.51	−0.14	0.05	0.885
7 to 9 h (normal)	7.18 (3.32)	0.42	0.51	0.83	0.04	0.405	0.23	0.51	0.45	0.02	0.653
More than 9 h	7.15 (3.76)	Ref.					Ref.				
Smoking											
Yes	6.80 (3.17)	Ref.					Ref.				
No	7.19 (3.21)	0.83	0.31	2.68	0.07	**0.008**	0.66	0.34	1.92	0.05	0.055
Daily newspaper reading/bulletin watching											
Yes	7.00 (3.32)	Ref.					Ref.				
No	7.19 (3.12)	0.24	0.25	0.96	0.02	0.336	0.17	0.25	0.66	0.01	0.511
Father’s occupation											
Job holder	7.36 (3.13)	0.92	0.68	1.35	0.09	0.178	0.74	0.69	1.08	0.08	0.281
Businessman	6.91 (3.27)	0.27	0.69	0.39	0.02	0.697	0.14	0.69	0.21	0.01	0.837
Others	6.81 (3.30)	0.09	0.72	0.13	<0.01	0.895	−0.16	0.73	−0.22	−0.01	0.824
Unemployed	7.02 (3.00)	Ref.					Ref.				
Self-perception about own physical health											
Good	7.12 (3.20)	0.87	0.37	2.35	0.06	**0.019**	0.80	0.37	2.16	0.05	**0.031**
Bad	7.07 (3.23)	Ref.					Ref.				

*B* = unstandardized regression coefficient; SE = Standard error; *β* = standardized regression coefficient; Bold indicates significant; ^†^F_(16,1,341)_ = 2.31; *p* < 0.001, *R*^2^_Adj_ = 0.0152 indicates that 1.53% of the variation of the dependent variable is explained by the independent variables in this regression model.

### 3.3 Perception toward linkage between dengue and climate change

The mean score for attitude items was 26.60 (SD = 4.12) out of 33, corresponding to an overall accuracy of 80.60%. According to multiple linear regression analysis, factors positively associated with attitude scores included: (i) having higher age (*ꞵ* = 0.08, *p* = 0.008), (ii) being unemployed (*ꞵ* = 0.10, *p* < 0.001) in reference to ‘employed’, (iii) having normal time of sleep (*ꞵ* = 0.05, *p* = 0.044), (iv) daily newspaper reading/ bulletin watching (*ꞵ* = 0.06, *p* = 0.024), (v) having good self-perception about own physical health (*ꞵ* = 0.08, *p* = 0.001) (Table 4).

**Table 4:** Regression analysis predicting perception about the relation between dengue and climate change.

Variables	Overall	Bivariable regression analysis					Multivariable regression analysis				
	Mean (SD)	*B*	SE	*t*	*β*	*p*-value	*B*	*SE*	*t*	*β*	*p*-value
Age		0.20	0.07	2.97	0.08	**0.003**	0.03	0.08	0.41	0.01	0.679
Education level											
Below university	26.91 (4.03)	Ref.					Ref.				
University	26.31 (4.20)	0.59	0.22	2.69	0.07	**0.007**	0.02	0.26	0.08	<0.01	0.934
Permanent Residence											
Rural	26.44 (4.17)	Ref.					Ref.				
Urban	26.61 (4.12)	0.17	0.25	0.68	0.03	0.497	0.19	0.30	0.65	0.02	0.518
Semi-urban	26.92 (4.02)	0.47	0.32	1.47	0.01	0.142	0.13	0.36	0.37	0.01	0.709
Monthly family income											
> 20,000 BDT	26.55 (4.03)	Ref.					Ref.				
20,000–30,000 BDT	26.65 (4.14)	0.09	0.25	0.39	0.01	0.699	−0.57	0.30	−1.87	−0.06	0.061
> 30,000 BDT	26.57 (4.23)	0.01	0.33	0.05	<0.01	0.960	−0.45	0.40	−1.12	−0.03	0.263
Gender											
Male	26.76 (4.39)	0.30	0.22	1.38	0.03	0.168	0.09	0.51	0.18	<0.01	0.858
Female	26.45 (4.83)	Ref.					Ref.				
Marital status											
Unmarried	26.62 (4.11)	0.17	0.42	0.41	0.01	0.679	−0.23	0.27	−0.85	−0.02	0.394
Married	26.45 (4.27)	Ref.									
Family type											
Nuclear	26.71 (4.25)	0.20	0.22	0.90	0.02	0.369	0.52	0.26	2.00	0.05	**0.046**
Large	26.51 (3.98)	Ref.					Ref.				
Employment status											
Employed	25.64 (4.36)	Ref.					Ref.				
Unemployed	26.81 (4.04)	1.16	0.29	3.97	0.10	**<0.001**	0.29	0.34	0.85	0.02	0.394
Previous history of dengue											
Yes	26.48 (4.19)	Ref.					Ref.				
No	26.65 (4.10)	0.16	0.26	0.64	0.01	0.521	0.23	0.37	0.63	−0.02	0.531
Family history of dengue											
Yes	26.55 (4.00)	Ref.					Ref.				
No	26.63 (4.17)	0.07	0.25	0.29	<0.01	0.769	−0.27	0.31	−0.86	−0.02	0.389
Previous history of vector borne disease											
Yes	26.52 (4.19)	Ref.					Ref.				
No	26.64 (4.10)	0.12	0.26	0.46	0.01	0.643	−0.35	0.36	−1.00	−0.03	0.319
Average sleeping time											
Less than 7 h	26.31 (3.99)	Ref.					Ref.				
7–9 h (normal)	26.78 (4.17)	0.46	0.23	2.01	0.05	**0.044**	0.30	0.26	1.16	0.03	0.245
More than 9 h	27.32 (4.50)	1.00	0.45	2.21	0.06	**0.027**	0.07	0.51	0.14	<0.01	0.888
Smoking											
Yes	26.21 (4.47)	Ref.					Ref.				
No	26.71 (4.02)	0.50	0.27	1.81	0.04	0.070	0.63	0.34	1.86	0.05	0.064
Daily newspaper reading/bulletin watching											
Yes	26.90 (4.18)	0.51	0.22	2.27	0.06	**0.024**	−0.18	0.25	−0.70	−0.01	0.485
No	26.39 (4.06)	Ref.					Ref.				
Father’s occupation											
Job holder	26.66 (4.13)	1.18	0.61	1.94	0.14	0.052	0.69	0.69	1.00	0.07	0.316
Businessman	26.60 (4.11)	1.13	0.61	1.83	0.12	0.068	0.15	0.69	0.22	0.01	0.826
Others	26.74 (3.88)	1.26	0.64	1.95	0.11	0.051	−0.16	0.73	−0.23	−0.01	0.819
Unemployed	25.47 (5.06)	Ref.					Ref.				
Self-perception about own physical health											
Good	26.75 (4.06)	1.09	0.32	3.32	0.08	**0.001**	0.85	0.37	2.28	0.06	**0.023**
Bad	26.66 (4.40)	Ref.					Ref.				

*B* = unstandardized regression coefficient; SE = Standard error; *β* = standardized regression coefficient; Bold indicates significant; ^†^F_(16,1,314)_ = 4.72; *p* < 0.001, *R*^2^_Adj_ = 0.042 indicates that 4.2% of the variation of the dependent variable is explained by the independent variables in this regression model.

### 3.4 Relationship between knowledge about climate change and perceptions regarding the link between dengue and climate change

As per as multiple linear regression analysis, higher knowledge about climate change was positively associated with higher perceptions regarding the link between dengue and climate change (*ꞵ* = 0.38, *p* < 0.001) (Table 5).

**Table 5:** Regression analysis to examine the relationship between knowledge about climate change and perceptions regarding the link between dengue and climate change.

Variables	Knowledge about climate change				
	*B*	SE	*t*	*ꞵ*	*p*-value
Perceptions regarding the link between dengue and climate change	0.43	0.02	15.44	0.38	**<0.001**

*B* = unstandardized regression coefficient; SE = Standard error; *β* = standardized regression coefficient; Bold indicates significant; ^†^F_(1,1,356)_ = 238.47; *p* < 0.001, *R*^2^_Adj_ = 0.149 indicates that 14.9% of the variation of the dependent variable is explained by the independent variable in this regression model.

## 4 Discussion

Climate change is a significant global concern in today’s world, and it has a noticeable impact on vector-borne diseases such as Dengue, which result in millions of deaths worldwide (9, 16). The study is of great significance, as it identifies critical variables that influence youth’s perception and knowledge. Key factors such as marital status, family structure, smoking habits, self-perception of physical health, sleep patterns, social media usage, age, employment status, media consumption, and their relationship with knowledge and perceptions regarding climate change and its link to dengue fever play a pivotal role. By focusing on these significant variables, the study provides a foundation for developing tailored climate change education and awareness programs, enhancing knowledge, and fostering positive attitudes among the youth in Bangladesh (28). This, in turn, contributes to improved public health and environmental resilience in the face of climate change challenges.

The study found that being unmarried was positively associated with higher knowledge scores. This suggests that unmarried individuals may have more time and inclination to acquire knowledge about climate change (29). This finding aligns with some previous studies that have shown a positive correlation between marital status and knowledge of climate change (29, 30). Unmarried individuals may have more time to engage in educational activities (31). Residing in a nuclear family was positively associated with higher knowledge scores. This implies that individuals from nuclear families may have more access to educational resources or discussions about climate change (25). A Previous Australian study also indicated that family dynamics and discussions play a role in shaping an individual’s environmental awareness (32).

Non-smokers had higher knowledge scores, suggesting that lifestyle choices, such as not smoking, may be associated with greater awareness of environmental issues (33). Environmentally conscious individuals often make constructive lifestyle choices, naturally giving priority to factors related to both the environment and health (34). While this specific variable may not be a common focus in previous research, lifestyle choices have been linked to environmental consciousness in various studies (34, 35).

Participants who had a good self-perception of their physical health had higher knowledge and perception scores. This could be due to the fact that individuals with better health perceptions are more conscious of their overall well-being, including environmental factors (36). Previous studies from United Kingdom and Poland have highlighted the connection between personal health and environmental awareness (37, 38). Those with regular sleep patterns demonstrated higher knowledge scores and perception toward linkage between Dengue and climate change. Adequate sleep may lead to better cognitive functioning, enabling individuals to absorb and retain knowledge more effectively (39). Similar to a study in Ghana (3), sleep quality and knowledge acquisition are recognized as interconnected factors (40).

Spending 2 to 4 h on social media was positively associated with higher knowledge scores. This finding suggests that moderate social media platforms can be effective tools for disseminating information about climate change (41). In line with a previous study in Poland (42), the role of social media in environmental education is a growing area of research (43).

Regarding the connection between Dengue and climate change, the study discovered that a higher age was associated with more favorable attitudes toward the correlation between Dengue and climate change which is in line with a previous research (44). Evidence shows older individuals tend to have a better understanding of complex issues like climate change (45). Additionally, the higher-level educational curriculum in Bangladesh includes information about climate change and Dengue, which can influence their opinions (46).

Being unemployed was positively associated with more favorable attitudes. Moreover, engaging in daily newspaper reading or bulletin watching was associated with more positive attitudes. Media consumption has often been linked to environmental awareness, as it provides individuals with information and perspectives on climate-related issues (47).

The study highlights a positive association between knowledge about climate change and attitudes regarding the link between dengue and climate change. It’s expected that individuals with a more comprehensive understanding of climate change would also have a better perception of linkage between Dengue and climate change (5). This relationship is well-supported by previous researches, which consistently demonstrates that a better understanding of climate change is linked to more informed and positive attitudes about its consequences (4, 48).

In summary, the significant variables identified in this study align with some aspects of previous research. However, the unique cultural and demographic context of Bangladeshi youth may lead to variations in the importance of these variables compared to studies in other regions. These findings emphasize the need for context-specific environmental education and awareness programs to address the unique factors influencing knowledge and perceptions in this population.

### 4.1 Strengths and limitations of the study

The main strength of this study lies in its thorough investigation into knowledge of climate change and attitudes toward dengue-climate connection, employing a sizable sample. This research marks a pioneering initiative targeting the youth demographic in Bangladesh. The results offer valuable insights to policymakers, facilitating the formulation of efficient strategies to tackle dengue outbreaks and preventive measures. The study possesses several limitations warranting acknowledgment. Primarily, the utilization of convenience sampling may introduce selection bias, thereby constraining the applicability of the findings to a more broader population compared to this. Secondly, the data collected may be susceptible to recall bias, response bias, and social desirability bias, potentially compromising response accuracy due to reliance on self-reported measures. Additionally, the cross-sectional nature of the study hinders the establishment of causal relationships and the comprehension of longitudinal changes. A longitudinal or prospective study would offer valuable insights in this regard. Furthermore, the absence of a comparison group and the limited generalizability to diverse regions or countries impose constraints on the broader applicability of the findings. Finally, positively framed questions of both the knowledge and attitudes may lead to biases. When interpreting the findings and forming conclusions, it is crucial to take into account these constraints.

## 5 Conclusion

In conclusion, this study highlights several significant factors influencing knowledge and perceptions regarding climate change and its link to Dengue among the youth in Bangladesh. The findings provide valuable insights for policymakers and interventions aimed at improving climate change awareness and fostering positive attitudes. Key factors include marital status, family structure, smoking habits, self-perception of physical health, sleep patterns, social media usage, age, employment status, and media consumption. Understanding these factors is essential for tailoring effective environmental education programs and increasing awareness among the youth, ultimately contributing to improved public health and environmental resilience in the face of climate change challenges.

## 6 Policy recommendations

- Roll out extensive public awareness initiatives aimed at informing the public about dengue fever, its transmission, and preventive strategies, including how climate change contributes to worsening dengue outbreaks.
- Encourage community participation in dengue prevention efforts through community clean-up drives, source reduction activities, and promoting the use of mosquito repellents and bed nets.
- Strengthen healthcare infrastructure, including increasing access to diagnostic and treatment facilities for dengue fever, especially in high-risk areas.
- Implement effective vector control programs, including regular larviciding and insecticide spraying in areas prone to dengue outbreaks.
- Utilize environmental management tactics to diminish mosquito breeding grounds, including effective waste disposal, upkeep of drainage systems, and urban design aimed at reducing stagnant water buildup.
- Develop climate resilience strategies that include dengue prevention measures, such as early warning systems for disease outbreaks and adaptive measures to mitigate the impact of climate change on dengue transmission.
- Allocate resources toward dengue research and surveillance systems to monitor disease patterns, pinpoint regions with elevated risk, and assess the efficiency of implemented control strategies.

## Data availability statement

The raw data supporting the conclusions of this article will be made available by the authors, without undue reservation.

## Ethics statement

The studies involving humans were approved by the Biosafety, Biosecurity and Ethical Clearance Committee, Jahangirnagar University, Savar., Dhaka-1342, Bangladesh [Ref. No: BBEC, JU/M2023/08(59)]. All procedures of the present study were conducted in accordance with human involving research guidelines (e.g., Helsinki declaration). Inform written consent was obtained from each participant where the study’s procedures, objectives, and confidentiality about their information, etc. were clearly documented. The data were collected anonymously and analyzed using numerical codes. The studies were conducted in accordance with the local legislation and institutional requirements. The participants provided their written informed consent to participate in this study. The animal study was approved by the Biosafety, Biosecurity and Ethical Clearance Committee, Jahangirnagar University, Savar., Dhaka-1342, Bangladesh [Ref. No: BBEC, JU/M2023/08(59)]. All procedures of the present study were conducted in accordance with human involving research guidelines (e.g., Helsinki declaration). Inform written consent was obtained from each participant where the study’s procedures, objectives, and confidentiality about their information, etc. were clearly documented. The data were collected anonymously and analyzed using numerical codes. The study was conducted in accordance with the local legislation and institutional requirements.

## Author contributions

AS: Conceptualization, Data curation, Formal analysis, Investigation, Methodology, Validation, Writing – original draft, Writing – review & editing. MH: Data curation, Formal analysis, Methodology, Writing – original draft. AA: Investigation, Validation, Writing – review & editing. MR: Writing – original draft, Writing – review & editing. MS: Supervision, Writing – review & editing.
